# Evaluation of the clinical cardiac safety of pemigatinib, a fibroblast growth factor receptor inhibitor, in participants with advanced malignancies

**DOI:** 10.1002/prp2.906

**Published:** 2021-12-24

**Authors:** Xiaohua Gong, Tao Ji, Xiang Liu, Xuejun Chen, Swamy Yeleswaram

**Affiliations:** ^1^ Incyte Research Institute Incyte Corporation Wilmington Delaware USA

**Keywords:** cardiac safety, early phase, oncology

## Abstract

Pemigatinib is a potent inhibitor of the fibroblast growth factor receptor (FGFR) family of receptors that is approved for the treatment of cholangiocarcinoma with *FGFR2* fusion or other rearrangements. Data from a first‐in‐human clinical study were used to assess the potential for pemigatinib to produce clinically significant effects on heart rate (HR) and cardiac repolarization (QTc). A central tendency analysis for electrocardiogram (ECG) outliers and a plasma concentration‐QTc analysis were conducted to assess cardiac safety in the first‐in‐human pemigatinib study (FIGHT‐101; NCT02393248). The study included 113 participants who received at least one dose of pemigatinib as monotherapy and had at least one pair of plasma pharmacokinetic (PK) and ECG data points collected. Timed 12‐lead ECGs were performed within 15 min of PK blood draws. The ECG parameters for each dose group in the study varied within expectations for patients with advanced malignancies. Categorical analysis of QT interval corrected for HR by Fridericia's method did not reveal dose dependence in the incidence of outliers, and the results of the central tendency and concentration‐QTc analyses did not suggest a dose‐ or concentration‐dependent drug effect. Least squares mean change from baseline in HR was small and did not indicate a clinically relevant effect on HR, and no effect was observed on cardiac conduction as assessed by PR and QRS intervals. In conclusion, pemigatinib does not exhibit any clinically significant prolongation of QTc or dose‐dependent changes in HR.

Clinical trial registration: ClinicalTrials.gov NCT02393248.

## Graphical abstract

Data from the first‑in‑human FIGHT‐101 (NCT02393248) study of pemigatinib in patients with advanced malignancies were used in a central tendency analysis to assess the potential for pemigatinib to produce clinically significant effects on heart rate and cardiac repolarization.

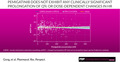

**Abbreviations**
BA: Bland–Altman
CI: confidence interval
ECG: electrocardiogram
ECG: electrocardiogram
FGFR: fibroblast growth factor receptor
GCP: Good Clinical Practices
HR: heart rate
ICH: International Council for Harmonisation
PK: pharmacokinetics
QTc: cardiac repolarization

**What is already known about this subject**

- Pemigatinib is a novel targeted therapy approved for treatment of cholangiocarcinoma with *FGFR2* fusion/rearrangements.
- It is important to understand the risk of cardiac toxicity (in particular, QTc prolongation) associated with non‐antiarrhythmic drugs.
- Early‐phase clinical studies are used to explore the potential for clinically relevant effects on QTc and heart rate.

**What this study adds**

- Pemigatinib was not associated with any clinically relevant effects on cardiac repolarization.
- There was no pemigatinib plasma concentration‐dependent increase in the change from baseline QT interval corrected for heart rate by Fridericia's method.

## 1 INTRODUCTION

Pemigatinib (INCB054828) is a potent and selective inhibitor of fibroblast growth factor receptor (FGFR)1, FGFR2 , and FGFR3 ^1^ that is under development for the treatment of malignant diseases or other diseases related to FGFR dysregulation. It is primarily metabolized by cytochrome P450 3A4 and exhibits low renal clearance and minimal renal excretion.^2^, ^3^ Pemigatinib was recently approved by the US Food and Drug Administration, the European Medicines Agency and the Japan Pharmaceuticals and Medical Devices Agency for the treatment of adults with previously treated, unresectable, locally advanced or metastatic cholangiocarcinoma with an FGFR2 gene fusion or other rearrangement.^2^, ^4^, ^5^

The International Council for Harmonisation (ICH) E14 guidance provides recommendations for assessing the potential of non‐antiarrhythmic drugs to delay cardiac repolarization.^6^ In accordance with these guidelines, the cardiac safety of pemigatinib was investigated during the drug development process. In nonclinical studies, pemigatinib did not have an effect on cardiac repolarization in in vitro and in vivo assays (unpublished data on file, Incyte Corporation). Assessment of human ether‐á‐go‐go channel interactions in clinical study participants showed a half maximal inhibitory concentration of >8 μM (the highest feasible concentration because of solubility), which is >300‐fold higher than the free mean maximum plasma drug concentration at the therapeutic dose of 13.5 mg (236 nM [total] or 22.2 nM [free]),^3^ suggesting low proarrhythmic activity. Based on these studies, pemigatinib is not expected to have a cardiac effect.

Concentration‐response analysis from data acquired during early clinical development can be used to evaluate the potential for clinically relevant effects of a drug on QT prolongation.^7^ This analysis of data from the first‐in‐human study FIGHT‐101 (NCT02393248) describes the relationship between pemigatinib plasma concentration and QTc interval, based on data from study participants receiving pemigatinib monotherapy at doses of 1 to 20 mg once daily (QD). The results of QT interval corrected for heart rate by Fridericia's method (QTcF) categorical and central tendency analyses, as well as the effect of pemigatinib on cardiac conduction (PR and QRS) and heart rate (HR), are also described.

## 2 MATERIALS AND METHODS

### 2.1 Ethics

The study protocols, protocol amendments, and consent forms were approved by qualified independent review boards/independent ethics committees before participant enrolment. All participants provided written informed consent before enrolling in the study. The study was conducted at 14 sites (13 in the United States and 1 in Denmark) in accordance with Good Clinical Practice guidelines and the ethical principles outlined in the Declaration of Helsinki.

### 2.2 Study design and populations

FIGHT‐101 (NCT02393248) was a first‐in‐human, open‐label, dose‐escalation and dose‐expansion study of the safety, tolerability, dose‐limiting toxicities, pharmacokinetics (PK), pharmacodynamics, and preliminary efficacy of pemigatinib in patients with advanced malignancies. The study population included male and female participants, 18 years of age or older, with any advanced solid tumor malignancy. Participants self‐administered doses of pemigatinib QD on either an intermittent schedule (2‐weeks‐on and 1‐week‐off) or a continuous administration regimen. For the purpose of analysis, the data for intermittent and continuous QD dosing of pemigatinib monotherapy treatments were combined, as the PK and electrocardiogram (ECG) assessments were collected prior to the first break in intermittent dosing (day 14).

The study was conducted in three parts. Part 1 was a monotherapy dose escalation of pemigatinib, in which doses of 1–20 mg QD (1, 2, 4, 6, 9, 13.5, and 20 mg) were administered on an intermittent or continuous schedule. Part 2 was a monotherapy dose expansion that evaluated doses that were selected based on evaluations in Part 1 (9, 13.5, and 20 mg) in participants with amplification, mutation, or translocation of *FGFR 1*, *2*, or *3*, or alteration of *FGF 1–23*. Part 3 involved dose‐finding to determine the recommended phase 2 dose of pemigatinib in combination with chemotherapy. Data collected from participants in Parts 1 and 2 of the study are included in the analyses reported here.

### 2.3 Pharmacokinetics

Plasma PK samples were collected predose and 0.5, 1, 2, 4, 6, and 8 h postdose on days 1 and 14 of the first cycle and predose only on days 2, 8, 15, and 16. Participants fasted for 8 h before taking study drug in the clinic and fasted for 1 additional hour after. The plasma samples were assayed by a validated liquid chromatography–tandem mass spectrometry method with a linear validated range of 1 to 1000 nM in human plasma. Standard noncompartmental PK methods were used to analyze pemigatinib plasma concentration versus time data using Phoenix^®^ WinNonlin^®^ version 8.0 (Certara USA Inc.).

### 2.4 Electrocardiogram assessments

The 12‐lead ECG recordings from 113 participants in Part 1 and Part 2 were assessed. Single ECG collection was initially planned for all visits and planned time points at predose and 1 and 2 h postdose; starting at higher doses (≥6 mg), triplicate ECGs were collected and a 4‐h postdose time point was added to the visit on the first day of cycle 14. As a result, most (90%) postbaseline raw ECG measurements were made in triplicate, especially for dose groups receiving ≥9 mg QD. All ECGs were performed after 5 min of semi‐recumbent rest. Timed 12‐lead ECGs were performed before and within 15 min of the PK blood draw at the corresponding time point. All 12‐lead ECGs were recorded by centrally provided equipment and analyzed at the central ECG laboratory using a semiautomated technique (i.e., over‐readings of ECG intervals). Machine‐generated ECG interval readings were analyzed together with over‐readings by the central laboratory for the ECG method bias analysis. ECG intervals were measured in a blinded manner by the core laboratory and the ECG database was locked before any statistical analysis was undertaken.

The baseline ECG was defined as the average of all ECGs measured prior to the first administration of study drug on the first day of the first cycle of treatment.

### 2.5 Data handling

#### 2.5.1 Electrocardiogram time points

Composite categorical time variables were derived (i.e., PK stage/time point) based on visit and nominal time point but with the steady‐state visits (day 8 and day 14 of the first cycle) combined.

#### 2.5.2 Time‐matched mapping between pharmacokinetics and electrocardiogram

For the purpose of time‐matched concentration‐QTcF (C‐QTcF) analysis, only the 1:1 data pairs of PK data and QTcF were included. Mapping between ECG and PK was based on the actual date/time of PK and ECG assessments.

### 2.6 Statistical methods

All statistical analyses were performed using the statistical software SAS^®^ version 9.4 (SAS Institute, Inc.).

#### 2.6.1 Categorical analysis

A participant or time point was determined as an outlier if the ECG intervals fell into the following categories (which were assessed separately): absolute QTcF values of >450 and ≤480 ms, >480 and ≤500 ms, or >500 ms; change from baseline (Δ)QTcF of >30 and ≤60 ms or ΔQTcF >60 ms; ΔPR >25% resulting in PR >200 ms; ΔQRS >25% resulting in QRS >120 ms; and HR changes reflecting either a >25% decrease from baseline to an HR of <50 bpm or a >25% increase from baseline to an HR of >100 bpm. All outliers were summarized for each treatment group on the basis of incidence rates. A participant was counted only once for a particular outlier event if the participant experienced more than one episode of that event.

#### 2.6.2 Central tendency analysis

The central tendency analysis for ΔQTcF (and other ΔECG intervals such as ΔHR, ΔPR, and ΔQRS) was based on a repeated measures model that had ΔQTc as the dependent variable, composite stage/time points and dose as categorical fixed effects and baseline QTcF as continuous fixed effects covariates. A compound symmetry covariance matrix was specified for the repeated measures at the composite stage/time points for participants due to the convergence challenges encountered with the otherwise default unstructured covariance matrix. From this analysis, the least squares (LS) mean, standard error, and two‐sided 90% confidence interval (CI) were calculated for each dose of pemigatinib and each stage/time point, separately.

#### 2.6.3 Concentration‐QTc analysis

QTcF was defined as:

$${\text{QTcF}}_{i,j}={\text{QT}}_{i,j}/{\text{RR}}_{i,j}^{1/3}$$

for participant *i* at time point *j*, in which the RR interval is in the unit of seconds. Change from baseline QTcF for participant *i* at time *j* was derived as:

$$\Delta {\text{QTc}}_{i,j}={\text{QTc}}_{i,j}‐{\text{QTc}}_{i0}$$

where QTcF_*i*0_ stands for the baseline QTcF for participant *i*. The other ΔECG parameters, such as ΔHR, ΔPR, and ΔQRS, were derived in the same way.

The relationship between pemigatinib plasma concentrations and ∆QTcF was investigated using the prespecified linear mixed‐effects modeling approach,^8^ which can be written as:

ΔQTcF=1+CONC+adjusted_baseline

with random effects of participants on both intercept and concentration. It was assumed that the random effects are normally distributed as a bivariate normal random variable with mean [0, 0] and a 2 × 2 unstructured covariance matrix G, whereas the residuals are independent and identically normally distributed with mean 0 and variance $\sigma_{e}^{2}$. Model parameters were estimated using the restricted maximum likelihood approach. The degrees of freedom estimates were determined by the Kenward–Roger method.^9^

An exploratory analysis was performed to assess key assumptions undertaken by the C‐ΔQTcF linear model. Change from baseline HR was assessed with mean increases or decreases >10 bpm to be considered problematic. Exploratory graphical analyses of the joint plot of time‐aligned LS mean of ΔQTcF and observed ΔQTcF for each postdose time point and the mean pemigatinib concentrations at the same time points were performed. The adequacy of using a linear model was assessed by the C‐ΔQTcF plot incorporating a trend line (e.g., locally estimated scatterplot smoothing [LOESS]^10^ and/or linear regression). Furthermore, the same linear mixed‐effects model, with the addition of a quadratic concentration term, was fitted and the quadratic term was tested at the two‐sided 5% significance level.

The following goodness‐of‐fit plots were assessed for the C‐ΔQTcF models: scatterplot of predicted ΔQTcF versus residuals, scatterplot of concentration versus residuals, scatterplot of baseline QTcF versus residuals, Q–Q plot of residuals, Q–Q plot of random effects and quantiles of concentrations and observed ΔQTcF overlaid with model‐predicted ΔQTcF.

The geometric mean of the steady‐state peak plasma concentration (C_max,ss_) values of pemigatinib for participants in each of the pemigatinib dose groups was retrieved from the PK analysis of FIGHT‐101.^3^ The predicted effect and its two‐sided 90% CI for ΔQTcF at the geometric mean *C*_max,ss_ were obtained for each pemigatinib dose separately. The mean and two‐sided 90% CI for ΔQTcF were computed using the ESTIMATE statement per SAS^®^ PROC MIXED.

#### 2.6.4 Method bias sensitivity analysis

In this analysis, the automatic machine readings of ECGs were compared with the results of cardiologists’ over‐readings at the ECG central laboratory, as described in Ferber et al.^11^ and Gong et al.^12^

### 2.7 Nomenclature of targets and ligands

Key protein targets and ligands in this article are hyperlinked to corresponding entries in http://www.guidetopharmacology.org, the common portal for data from the IUPHAR/BPS Guide to PHARMACOLOGY,^13^ and are permanently archived in the Concise Guide to PHARMACOLOGY 2021/22.^14^

## 3 RESULTS

### 3.1 Demographics

One hundred sixteen participants with advanced malignancies were enrolled in the first‐in‐human study and received at least one dose of pemigatinib as monotherapy. Of these, 113 participants enrolled in Part 1 or Part 2 of this study had at least one pair of PK‐ECG data points collected (the ECG or PK/QTc population). Of the participants in this analysis population, 69 (61.1%) were women and the median age was 57 years (Table 1). In general, the ECG parameters at baseline in each dose group varied within expectations for small groups of patients with advanced malignancies. Mean absolute QTcF intervals at baseline ranged from 400 to 427 ms.

**TABLE 1:** Age, sex, and baseline ECG parameters (±SD) in the analysis population

Number of participants	Male	Female	Age, years	Baseline HR, bpm	Baseline QTcF, ms	Baseline PR, ms)^a^	Baseline QRS, ms
113	44 (38.9)	69 (61.1)	57 ± 13	75.2 ± 12.9	416.8 ± 17.4	157.4 ± 22.3	88.5 ± 11.1

Note

Male and female participants are reported as *n* (%). Data for all other categories are reported as mean ± SD.

Abbreviations: bpm, beats per minute; ECG, electrocardiogram; HR, heart rate; QTcF, QT interval corrected for heart rate by Fridericia's method; SD, standard deviation.

^a^ For baseline PR, *n* = 112.

### 3.2 QTcF categorical and central tendency analyses

Assessment of absolute QTcF and ∆QTcF outliers did not reveal dose dependence in the incidence of outliers (Table S1). Across all dose groups, no participant had absolute QTcF >500 ms. Two participants (one each in the 13.5‐mg and 20‐mg dose groups) had absolute QTcF between 480 and 500 ms, and 18 participants (4 [19.0%] in the 9‐mg QD dose group, 11 [16.4%] in the 13.5‐mg QD dose group and 3 [16.7%] in 20‐mg QD dose group) had absolute QTcF between 450 ms and 480 ms. In the change from baseline analysis, there was no incidence of ∆QTcF >60 ms across all dose groups. Nine participants (two in the 9‐mg QD dose group, six in the 13.5‐mg QD dose group and one in the 20‐mg QD dose group) had ∆QTcF between 30 and 60 ms.

The pattern of LS mean changes in QTcF interval across escalating doses was not consistent with a dose‐dependent drug effect. In the central tendency analysis, the LS means of ∆QTcF with respect to the nominal time were between −3 and 12 ms across dose groups that had more than one participant (≥6 mg QD) (Figure S1A). On day 1 of the first cycle, the largest LS mean ∆QTcF was observed at 1 h postdose for both the 9‐mg dose (8.9 ms; 90% CI: 4.69–13.06; *n* = 21) and the 13.5‐mg dose (7.8 ms; 90% CI: 5.42–10.15; *n* = 66). The LS mean ∆QTcF was 0.0 ms for the 20‐mg dose group at both 1 h (90% CI: −4.49 to 4.58; *n* = 18) and 2 h (90% CI: −4.56 to 4.50; *n* = 18) postdose. At steady state, the largest LS mean ∆QTcF observed for each dose was 11.8 ms (90% CI: 7.29–16.40; *n* = 17) at 1 h postdose for the 9‐mg dose group, 4.6 ms (90% CI: 2.02–7.12; *n* = 54) at 1 h postdose for the 13.5‐mg dose group and 3.8 ms (90% CI: −1.20 to 8.80; *n* = 14) at 2 h postdose for the 20‐mg dose group.

The LS means of ∆HR were all within ±10 bpm with respect to the nominal stage/time points for all dose groups (Figure S1B). These results suggest that pemigatinib had no drug effect on HR, and QTcF was the primary QTc endpoint.

### 3.3 Effect on cardiac conduction

At the studied doses, pemigatinib did not have a relevant effect on cardiac conduction as assessed by PR and QRS intervals. No participants had an increase in PR that represented a >25% increase from baseline to a PR interval of 200 ms, and only one participant (in the 13.5‐mg QD dose group) had QRS increased >25% from the baseline (105 ms) to a QRS of >120 ms.

In general, the LS mean ∆PR varied between −3 and 4 ms across doses ≥6 mg QD. Exceptions were seen in the 6‐mg QD dose group at 1 and 2 h postdose on day 1 of the first cycle, including findings of 5.2 ms (90% CI: −2.25 to 12.55; *n* = 4) and 12.9 ms (90% CI: 5.50–20.30; *n* = 4); these findings were not correlated with dose (Figure S1C).

LS mean ∆QRS values were all small (within ±5 ms) across doses ≥6 mg QD, except for a finding of 5.4 ms (90% CI: 1.20–9.57; *n* = 4) at 1 h postdose at steady state for the 6‐mg QD dose group (Figure S1D).

### 3.4 Concentration‐QTcF analysis

The clinical PK of pemigatinib in the first‐in‐human study have been reported, including mean plasma drug concentration‐time profiles of pemigatinib for each dose group.^3^ Pemigatinib is absorbed rapidly and typically attains peak concentration in plasma between 1 and 2 h postdose, after which the plasma concentration declines in a biexponential manner with a geometric mean terminal half‐life of 15.4 h.

**FIGURE 1:**
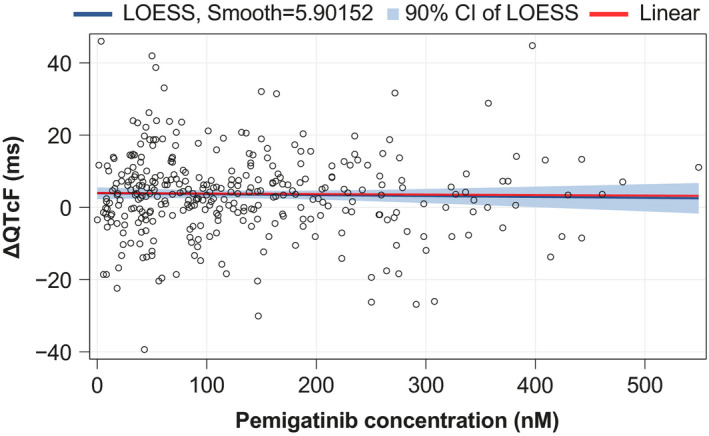
Scatterplot of observed pemigatinib plasma concentrations and ΔQTcF overlaid with a LOESS line and 90% CI. CI, confidence interval; LOESS, locally estimated scatterplot smoothing; ΔQTcF, change from baseline QT interval corrected for heart rate by Fridericia's method

When the mean pemigatinib plasma concentration, the mean observed ∆QTcF and LS mean ∆QTcF were plotted over time for dose groups of more than one participant (≥6 mg QD), no time‐delayed effects of pemigatinib exposures on QTcF were apparent (data not shown). A linear C‐QTcF relationship was assessed using a scatterplot of observed ∆QTcF and plasma pemigatinib concentrations overlaid with both a simple linear regression line and a LOESS regression line (Figure 1). The LOESS regression line did not suggest nonlinearity, and a linear model seemed acceptable.

A second assessment of the appropriateness of a linear relationship was used to test the inclusion of a quadratic term of plasma pemigatinib concentration, as a fixed effect term, into the model. The coefficient parameter associated with the quadratic concentration term was estimated as 0.000016 ms per (nM*nM) (95% CI: −0.00008 to 0.000116) with a *p* value of .7593, indicative of the lack of statistical support for inclusion of a quadratic concentration term into the model.

**FIGURE 2:**
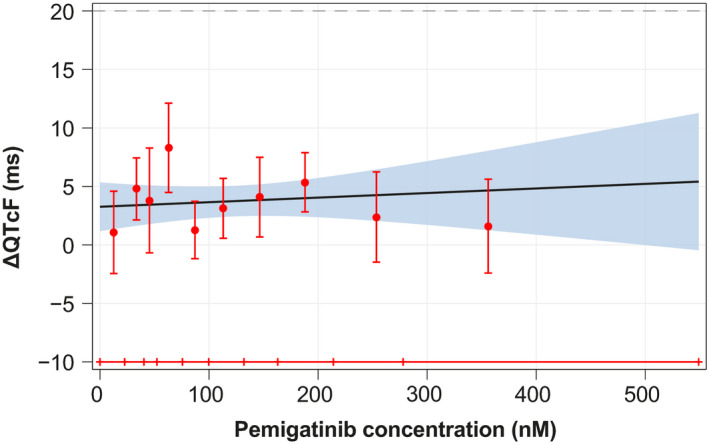
Model‐predicted ΔQTcF (mean and 90% confidence interval) overlaid with observed mean ΔQTcF (mean and 90% confidence interval) across deciles of pemigatinib plasma concentrations. ΔQTcF, change from baseline in QT interval corrected for heart rate by Fridericia's method

In the goodness‐of‐fit plot of the observed mean ΔQTcF (90% CI) within each pemigatinib concentration decile and the model‐predicted mean ΔQTcF (90% CI) (Figure 2), the predicted ΔQTcF values were relatively close to the observed values and the observed mean ΔQTcF did not show nonlinearity over the pemigatinib concentration deciles. This finding suggests that the prespecified C‐QTcF model provides a reasonable representation of the relationship between ΔQTcF and pemigatinib concentrations.

The estimated population slope of the C‐QTcF relationship was shallow (0.00391 ms per nM [95% CI: −0.01244 to 0.02026]), which was not different from 0 under a statistical significance level of α = .05.

The C‐QTcF model was used to predict the mean ΔQTcF at the geometric mean *C*_max,ss_ of each dose group. The ∆QTcF was predicted to be 3.36 ms (90% CI: 1.52–5.20) at the observed geometric mean *C*_max,ss_ (26.2 nM) after repeat doses of the lowest dose level studied (1 mg QD); 4.18 ms (90% CI: 2.13–6.24) at the observed geometric mean *C*_max,ss_ (236 nM [56.4% CV]) after repeat doses of the clinical therapeutic dose (13.5 mg QD); and 4.91 ms (90% CI: 0.60–9.22) at the observed geometric mean *C*_max,ss_ (421 nM [38.7% CV]) after repeat doses of the highest dose level studied (20 mg QD). At no dose level did the upper limit of the two‐sided 90% CI of the predicted ΔQTcF exceed 12 ms across the observed range of pemigatinib concentrations in this study (i.e., up to 549 nM).

In a sensitivity analysis, the same linear mixed‐effects C‐ΔQTcF model was fitted to the data on day 1 of the first cycle only. A total of 331 time‐matched C‐QTc observations were included in the whole analysis dataset, with 221 observations included in the subset of day 1 of the first cycle only. A goodness‐of‐fit plot showed robust alignment between the model‐predicted QT effects (mean ΔQTcF and 90% CI) and the observed ΔQTcF across pemigatinib concentration deciles (Figure S2). Across the observed range of pemigatinib concentrations on day 1 of the first cycle, the upper limit of the two‐sided 90% CI of the predicted ΔQTcF did not exceed 10 ms; thus, a large effect (i.e., >20 ms) on QTc interval can be excluded based on ICH E14 guidance.^7^

### 3.5 Electrocardiogram bias analysis

Comparison between fully automated machine readings of QTcF and the corresponding cardiologists’ over‐readings demonstrated minimal bias between the two ECG methods on the same set of ECG charts recorded from participants in Part 1 and Part 2 of the study. To analyze bias by dose group, the low dose levels of 1, 2, 4, 6, and 9 mg QD were combined (categorized as ≤9 mg QD) to overcome the small sample sizes. In all dose groups, the Bland–Altman (BA) slopes^15^ were between 0.03 and 0.05. For the pooled overall comparison, the mean BA slope was 0.04 (95% CI: 0.025–0.063), which corresponded to 4.0 ms over a QTcF range of 100 ms. All of the BA slopes were slightly positive and within the suggested threshold of 0.10 in the absolute scale.^11^

## 4 DISCUSSION

Current ICH E14 guidelines support the use of ECG data from early clinical development to detect small changes in cardiac repolarization as an assessment of the potential proarrhythmic effects of a drug.^6^, ^7^ The criteria for demonstrating that a new drug does not cause ECG effects of clinical concern include careful collection of ECGs, data analysis of ECGs with matched plasma concentration (C‐QTc) and sufficiently high plasma concentrations of the drug achieved at the studied doses. In Parts 1 and 2 of FIGHT‐101, pemigatinib was studied as monotherapy over a wide range of doses (between 1 and 20 mg QD) in 113 participants with advanced malignancies. The majority of postbaseline ECG measurements were collected in triplicate and paired with PK plasma sample collection within 15 min. The pemigatinib geometric mean *C*_max,ss_ for the recommended phase 2 dose (13.5 mg QD) and the highest dose evaluated (20 mg QD) was 236 nM (range: 77.3–660 nM) and 421 nM (range: 239–858 nM), respectively.^3^ In addition, the post hoc Bayesian estimated geometric mean *C*_max,ss_ at the therapeutic dose of 13.5 mg QD in the phase 2 pivotal trial FIGHT‐202 (INCB 54828‐202) was 185 nM (49.3–492 nM). Based on the satisfaction of criteria for early‐phase studies, the analyses in this study are appropriate to assess the potential of pemigatinib to produce clinically relevant cardiac effects.

The QTcF categorical analysis did not reveal dose dependence in the incidences of QTcF outliers. The LS means for ∆QTcF from the central tendency analysis were between −3 and 12 ms across dose groups that included more than one participant (≥6 mg QD). The largest LS mean ∆QTcF across these dose groups on day 1 of the first cycle was 8.9 ms (90% CI: 4.69–13.06; *n* = 21) at 1 h postdose at 13.5 mg QD; at steady state, the largest LS mean ∆QTcF was 11.8 ms (90% CI: 7.29–16.40; *n* = 17) at 1 h postdose at 9 mg QD. Both values were well below the threshold for large QT effects (>20 ms).

In the C‐QTcF analysis, a prespecified linear mixed‐effects model was determined to be an appropriate fit to the data and was used to establish the relationship between plasma pemigatinib concentration and ΔQTcF. The estimated slope of the C‐QTcF relationship was shallow and not significantly different from 0. Using this C‐QTcF model, a large QT/QTc effect exceeding the threshold of concern (20 ms) could be excluded within the observed range of pemigatinib plasma concentrations (up to 549 nM). In a sensitivity analysis, the same exposure‐response analysis was conducted using data from day 1 of the first cycle only, which accounts for approximately two‐thirds of total C‐QTcF data. The sensitivity analysis yielded similar results, supporting a lack of large effect on QTc interval at the maximum therapeutic dose level.

The ECG method bias sensitivity test estimated the mean BA slope as 0.04 (90% CI: 0.025–0.063) in the pooled data; this value was below the threshold of 0.10,^11^ suggesting the lack of a significant trend in the differences between QTcF intervals measured by the central laboratory and by the machine readings.

To further evaluate events that could signal potential proarrhythmic effects of pemigatinib, an analysis of treatment‐emergent adverse events of Torsade de pointes/QT prolongation (based on standardized Medical Dictionary for Regulatory Activities queries) or seizure was performed using the clinical database of pooled data from participants with advanced malignancies receiving pemigatinib monotherapy (466 participants in five studies). The results of the safety analysis supported the conclusion of no meaningful effect of pemigatinib on ECG parameters (unpublished data on file, Incyte Corporation).

Pemigatinib at doses up to 20 mg QD did not have a clinically relevant effect on cardiac conduction. No significant relationship between pemigatinib plasma concentration and change in QTcF was determined. A large QT/QTc effect exceeding the threshold of concern (20 ms) can be excluded within the observed range of pemigatinib plasma concentrations.

## DISCLOSURE

X.G., T.J., X.L., X.C., and S.Y. are employees of Incyte Corporation and own stock in Incyte.

## AUTHOR CONTRIBUTIONS

T.J., X.C., and S.Y were involved in research design. T.J. and X.C were involved in research performance. X.G., T.J., and X.L were involved in data analysis. All authors were involved in drafting the manuscript and critical revision of the manuscript.

## ETHICS APPROVAL STATEMENT

The study protocols, protocol amendments, and consent forms were approved by qualified independent review boards/independent ethics committees before participant enrolment. The study was conducted in accordance with Good Clinical Practice guidelines and the ethical principles outlined in the Declaration of Helsinki.

## PATIENT CONSENT STATEMENT

Written informed consent was obtained from all individual participants involved in the study.

## PERMISSION TO REPRODUCE MATERIAL FROM OTHER SOURCES

Not applicable.

## Supporting information

Supplementary Material

## Data Availability

Incyte Corporation (Wilmington, DE, USA) is committed to data sharing that advances science and medicine while protecting patient privacy. Qualified external scientific researchers may request anonymized datasets owned by Incyte for the purpose of conducting legitimate scientific research. Researchers may request anonymized datasets from any interventional study (except phase 1 studies) for which the product and indication have been approved on or after January 1, 2020 in at least one major market (eg, US, EU, JPN). Data will be available for request after the primary publication or 2 years after the study has ended. Information on Incyte's clinical trial data sharing policy and instructions for submitting clinical trial data requests are available at: https://www.incyte.com/Portals/0/Assets/Compliance%20and%20Transparency/clinical‐trial‐data‐sharing.pdf?ver=2020‐05‐21‐132838‐960.

## References

[1] Liu PCC , Koblish H , Wu L , et al. INCB054828 (pemigatinib), a potent and selective inhibitor of fibroblast growth factor receptors 1, 2, and 3, displays activity against genetically defined tumor models. PLoS One. 2020;15:e0231877. 32315352 10.1371/journal.pone.0231877 PMC7313537

[2] PEMAZYRE™ (pemigatinib) tablets, for oral use [prescribing information]. Incyte Corporation. April 2020. Accessed April 8, 2021. https://www.pemazyre.com/pdf/prescribing‐information.pdf

[3] Ji T , Lihou C , Asatiani E , et al. Pharmacokinetics and pharmacodynamics of pemigatinib, a potent and selective inhibitor of FGFR 1, 2, and 3, in patients with advanced malignancies. Mol Cancer Ther. 2019;18(12 Suppl):Abstract C071.

[4] Pemazyre: European Public Assessment Report – Summary of Product Characteristics. Accessed May 28, 2021. https://www.ema.europa.eu/en/documents/product‐information/pemazyre‐epar‐product‐information_en.pdf

[5] Incyte announces approval of Pemazyre^®^ (pemigatinib) in Japan for the treatment of patients with unresectable biliary tract cancer (BTC) with a fibroblast growth factor receptor 2 (FGFR2) fusion gene, worsening after cancer chemotherapy [press release]. Wilmington, DE: Incyte Corporation; March 23, 2021. Accessed July 13, 2021. https://investor.incyte.com/press‐releases/press‐releases/2021/Incyte‐Announces‐Approval‐of‐Pemazyre‐pemigatinib‐in‐Japan‐for‐the‐Treatment‐of‐Patients‐with‐Unresectable‐Biliary‐Tract‐Cancer‐BTC‐with‐a‐Fibroblast‐Growth‐Factor‐Receptor‐2‐FGFR2‐Fusion‐Gene‐Worsening‐After‐Cancer‐Chemotherapy/default.aspx

[6] FDA Guidance for Industry: E14 Clinical evaluation of QT/QTc interval prolongation and proarrhythmic potential for non‐antiarrhythmic drugs. October 2005. Accessed April 7, 2021. https://www.fda.gov/media/71372/download

[7] FDA Guidance for Industry: E14 Clinical evaluation of QT/QTc interval prolongation and proarrhythmic potential for non‐antiarrhythmic drugs – questions and answers (R3), 2015. Published June 2017. Accessed April 7, 2021. https://www.fda.gov/regulatory‐information/search‐fda‐guidance‐documents/e14‐clinical‐evaluation‐qtqtc‐interval‐prolongation‐and‐proarrhythmic‐potential‐non‐antiarrhythmic‐1

[8] Garnett C , Bonate PL , Dang Q , et al. Scientific white paper on concentration‐QTcF modeling. J Pharmacokinet Pharmacodyn. 2018;45:383‐397. Erratum in *J Pharmacokinet Pharmacodyn*. 2018;45:399. 29209907 10.1007/s10928-017-9558-5

[9] Kenward MG , Roger JH . Small sample inference for fixed effects from restricted maximum likelihood. Biometrics. 1997;53:983‐997. 9333350

[10] Cleveland WS . Robust locally weighted regression and smoothing scatterplots. J Am Stat Assoc. 1979;74:829‐836.

[11] Ferber G , Zhou M , Dota C , et al. Can bias evaluation provide protection against false‐negative results in QT studies without a positive control using exposure‐response analysis? J Clin Pharmacol. 2017;57:85‐95. 27271102 10.1002/jcph.779

[12] Gong X , Darpo B , Xue H , et al. Evaluation of clinical cardiac safety of itacitinib, a JAK1 inhibitor, in healthy participants. Clin Pharmacol Drug Dev. 2020;9:677‐688. 31821750 10.1002/cpdd.758

[13] Harding SD , Sharman JL , Faccenda E , et al. The IUPHAR/BPS Guide to PHARMACOLOGY in 2019: updates and expansion to encompass the new guide to IMMUNOPHARMACOLOGY. Nucleic Acids Res. 2018;46:D1091‐D1106. doi:10.1093/nar/gkx1121 29149325 PMC5753190

[14] Alexander SPH , Kelly E , Mathie A , et al. The Concise Guide to Pharmacology 2021/22: catalytic receptors. Br J Pharmacol. 2021;178:S264‐S312. 34529829 10.1111/bph.15541

[15] Bland JM , Altman DG . Comparing methods of measurement: why plotting difference against standard method is misleading. Lancet. 1995;346:1085‐1087. 7564793 10.1016/s0140-6736(95)91748-9

