# Supplementary material for: Evaluation of the clinical cardiac safety of pemigatinib, a fibroblast growth factor receptor inhibitor, in participants with advanced malignancies
Source: Pharmacol Res Perspect. 2021 Dec 24;10(1):e00906. doi: 10.1002/prp2.906 (PMC8929369; doi:10.1002/prp2.906)
Supplement: Supplementary file 1 — Supplementary Material [file PRP2-10-e00906-s001.pdf]

SUPPORTING INFORMATION

Evaluation of the clinical cardiac safety of pemigatinib, a fibroblast growth factor receptor inhibitor, in participants with advanced malignancies

Xiaohua Gong | Tao Ji | Xiang Liu | Xuejun Chen | Swamy Yeleswaram

Incyte Research Institute, Incyte Corporation, Wilmington, DE 19803, USA

Correspondence: Xiaohua Gong, PhD, Incyte Research Institute. Wilmington, DE 19803, USA.
E-mail: xgong@incyte.com.

TABLE S1 Categorical analysis of absolute or change from baseline QTcF

	Category	1/2/4/6 mg QD	9 mg QD	13.5 mg QD	20 mg QD	Total
Participant	QTcF total, N	7	21	67	18	113
	QTcF >450 and ≤480 ms, n (%)	0	4 (19.0)	11 (16.4)	3 (16.7)	18 (15.9)
	QTcF >480 and ≤500 ms, n (%)	0	0	1 (1.5)	1 (5.6)	2 (1.8)
	QTcF >500 ms, n (%)	0	0	0	0	0
Time point	QTcF total, N	47	129	387	119	688
	QTcF >450 and ≤480 ms, n (%)	0	11 (8.5)	28 (7.2)	8 (6.7)	47 (6.8)
	QTcF >480 and ≤500 ms, n (%)	0	0	1 (0.3)	1 (0.8)	2 (0.3)
	QTcF >500 ms, n (%)	0	0	0	0	0
Participant	Total, N	7	21	67	18	113
	ΔQTcF >30 and ≤60 ms, n (%)	0	2 (9.5)	6 (9.0)	1 (5.6)	9 (8.0)
	ΔQTcF >60 ms, n (%)	0	0	0	0	0
Time point	Total	40	107	316	101	564
	ΔQTcF >30 and ≤60 ms, n (%)	0	3 (2.8)	6 (1.9)	2 (2.0)	11 (2.0)
	ΔQTcF >60 ms, n (%)	0	0	0	0	0

QD = once daily; QTcF = QT interval corrected for heart rate by Fridericia's method.

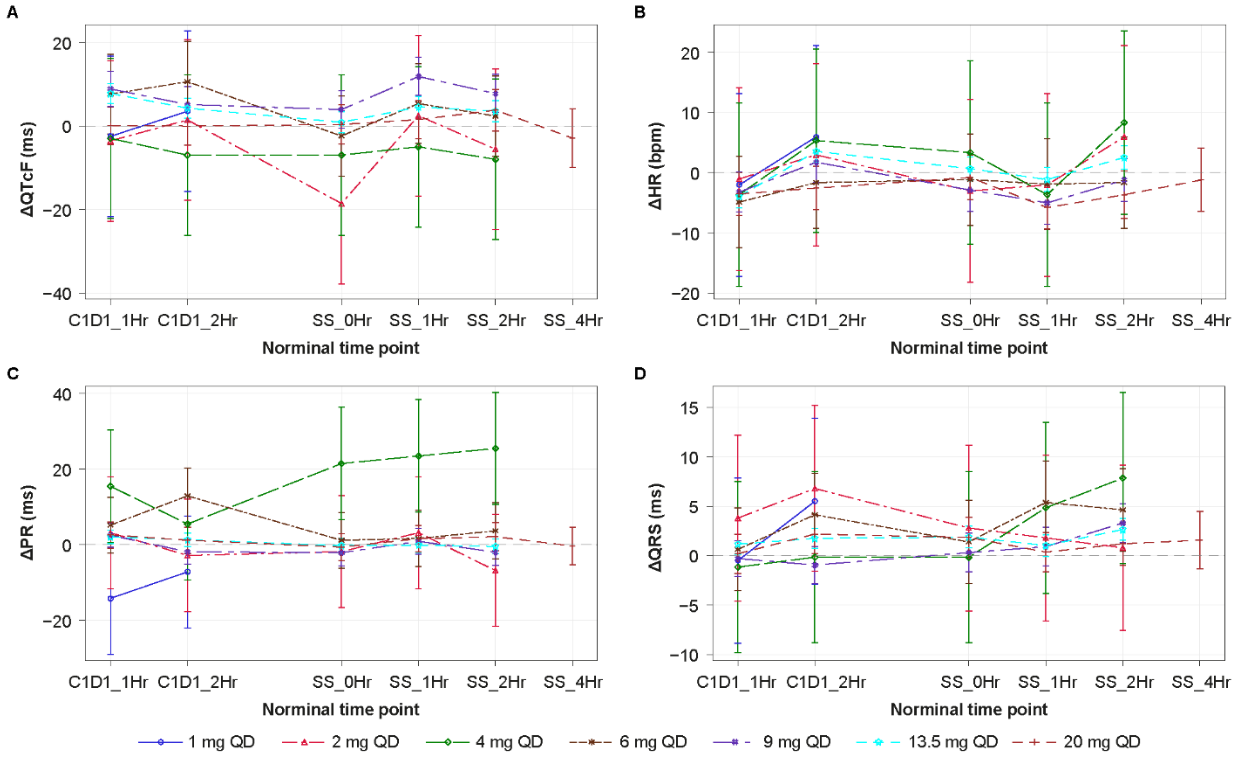

FIGURE S1 Change from baseline across nominal stage/time points for ECG parameters. Data shown are least squares mean ΔQTcF (A), ΔHR (B), ΔPR (C) and ΔQRS (D); bars represent 90% confidence intervals. Δ = change from baseline; C = cycle; D = day; ECG = electrocardiogram; Hr = hour; HR = heart rate; QD = once daily; QTcF = QT interval corrected for heart rate by Fridericia's method; SS = steady state.

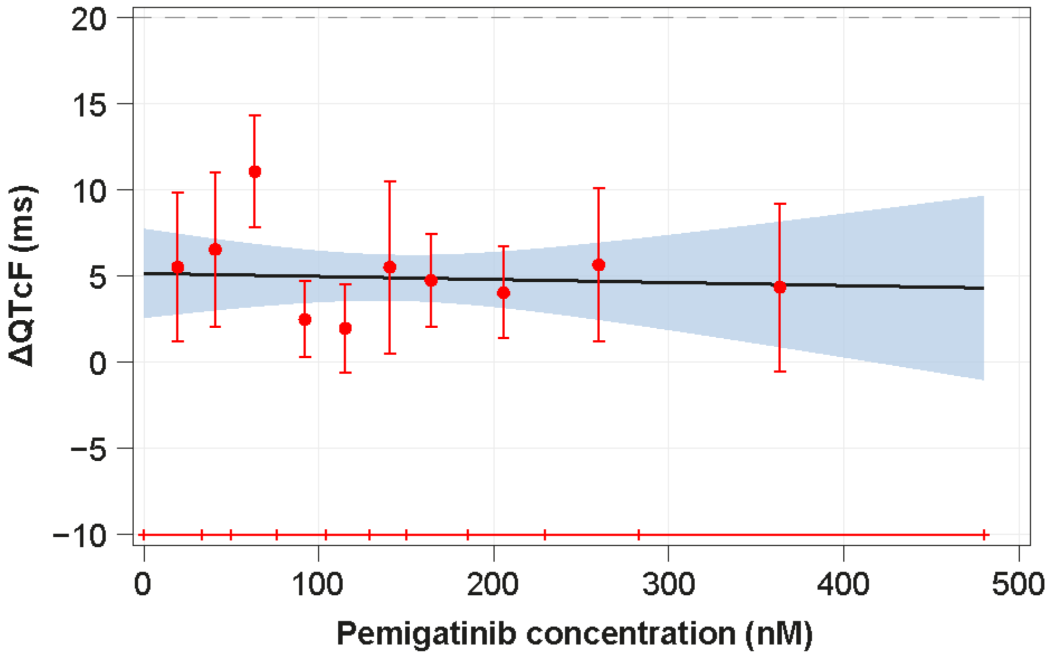

FIGURE S2 Model-predicted ΔQTcF (mean and 90% confidence interval) for day 1 of the first cycle overlaid with observed mean ΔQTcF (mean and 90% confidence interval) across deciles of pemigatinib plasma concentrations. ΔQTcF = change from baseline QT interval corrected for heart rate by Fridericia's method.
